# Effects of 30% vs. 60% inspired oxygen fraction during mechanical ventilation on postoperative atelectasis: a randomised controlled trial

**DOI:** 10.1186/s12871-023-02226-6

**Published:** 2023-08-08

**Authors:** Zhaoshun Jiang, Songbin Liu, Lan Wang, Wanling Li, Cheng Li, Feifei Lang, Ruoxi Li, Yue Zhou, Jiajun Wu, Yuxi Cai, Wen Xu, Zhen Chen, Zhijun Bao, Ming Li, Weidong Gu

**Affiliations:** 1 Department of Anesthesiology, Huadong Hospital Affiliated to Fudan University, Shanghai, China; 2 Shanghai Key Laboratory of Clinical Geriatric Medicine, Shanghai, China; 3 Department of General surgery, Shanghai XuHui Central Hospital, Shanghai, China; 4 Department of Radiology, Huadong Hospital Affiliated to Fudan University, Shanghai, China; 5 Department of Geriatric Medicine, Huadong Hospital Affiliated to Fudan University, Shanghai, China; 6 Research Centre on Aging and Medicine, Fudan University, Shanghai, China; 7 Department of Surgical Intensive Care Unit, Huadong Hospital Affiliated to Fudan University, Shanghai, China

**Keywords:** Atelectasis, Computed tomography, Fraction of inspired oxygen, Mechanical ventilation, Postoperative pulmonary complications

## Abstract

**Background:**

There is the ongoing debate over the effect of inspired oxygen fraction (FiO_2_) during mechanical ventilation on postoperative atelectasis. We aimed to compare the effects of low (30%) and moderate (60%) FiO_2_ on postoperative atelectasis. The hypothesis of the study was that 30% FiO_2_ during mechanical ventilation could reduce postoperative atelectasis volume compared with 60% FiO_2_.

**Methods:**

We performed a randomized controlled trial with 120 patients. Subjects were randomly assigned to receive 30% or 60% FiO_2_ during mechanical ventilation in a 1:1 ratio. The primary outcome was the percentage of postoperative atelectasis volume in the total lung measured using chest CT within 30 min after extubation. The secondary outcomes included different aeration region volumes, incidence of clinically significant atelectasis, and oxygenation index.

**Results:**

In total, 113 subjects completed the trial, including 55 and 58 subjects in the 30% and 60% FiO_2_ groups, respectively. The percentage of the postoperative atelectasis volume in the 30% FiO_2_ group did not differ from that in the 60% FiO_2_ group. Furthermore, there was no significant difference in the atelectasis volume between the two groups after the missing data were imputed by multiple imputation. Additionally, there were no significant differences in the volumes of the over-aeration, normal-aeration, and poor-aeration regions between the groups. No significant differences in the incidence of clinically significant atelectasis or oxygenation index at the end of surgery were observed between the groups.

**Conclusions:**

Compared with 60% FiO_2_, the use of 30% FiO_2_ during mechanical ventilation does not reduce the postoperative atelectasis volume.

**Trial registration:**

Chinese Clinical Trial Registry (http://www.chictr.org.cn). Identifier: ChiCTR1900021635. Date: 2 March 2019. Principal invetigator: Weidong Gu.

## Background

Postoperative atelectasis diagnosed by CT occurs in 60–90% of patients with mechanical ventilation under general anesthesia [1–4]. The postoperative atelectasis is associated with prolonged hospitalization, increased hospital costs, and increased postoperative 90-day mortality [5–7]. Therefore, prevention of postoperative atelectasis is important for perioperative management, especially in patients undergoing major surgery.

Mechanical ventilation provides the necessary oxygen supply for patients under general anesthesia during surgery, however, the optimal inspired oxygen fraction (FiO_2_) during mechanical ventilation remains controversial. The World Health Organization (WHO) recommends high FiO_2_ to reduce the risk of postoperative surgical site infections in patients undergoing general anesthesia [8]. However, this recommendation has sparked debate on the benefits and harms of hyperoxia [9, 10]. High oxygen concentrations have been reported to be associated with postoperative pulmonary complications (PPCs), especially atelectasis [11]. Kim et al. found that postoperative atelectasis occurred more frequently with 100% FiO_2_ than with 40% FiO_2_ [12]. And a meta-analysis found that the extent of postoperative atelectasis was more severe in the high intraoperative FiO_2_ group compared with the low FiO_2_ group [13]. Conversely, two randomised controlled trials have shown no differences in the incidence of PPCs, including atelectasis, between 80% and 30% FiO_2_ [3, 14].

Previous studies have provided conflicting results; thus the effect of FiO_2_ on atelectasis requires further investigation. Additionally, several issues in such research should be noted and improved upon. First, most of the previous studies usually compared the effects of extremely high FiO_2_ (80–100%) and low FiO_2_ (30–40%) on atelectasis. In clinical practice, a moderate FiO_2_ of 50–60% is more conventionally used. However, the effect of low (30%) versus moderate (60%) FiO_2_ on atelectasis remains unclear. Second, positive end-expiratory pressure (PEEP) and recruitment maneuvers have often been used in studies to investigate the effect of FiO_2_ on atelectasis [15, 16]. As PEEP and recruitment maneuvers could reduce the incidence and extent of atelectasis [17], the independent effect of FiO_2_ on atelectasis remains to be investigated. Third, although lung ultrasound was used in previous studies to diagnose atelectasis, it cannot measure the volume of atelectasis [16]. Computed tomography (CT) is the current gold standard for diagnosing atelectasis and it can accurately measure the volume of different aeration regions [18].

Therefore, we conducted a randomised controlled study to investigate the effects of 30% versus 60% FiO_2_ without PEEP and recruitment maneuvers on postoperative atelectasis volume measured by CT scans. We tested the hypothesis that 30% FiO_2_ during mechanical ventilation could reduce postoperative atelectasis volume compared with 60% FiO_2_.

## Methods

### Ethics

This prospective, randomized study was conducted from April 2019 to September 2020 at the Huadong Hospital affiliated to Fudan University, Shanghai, China. The study was appoved on 6 March 2019 by the Ethics Commission of Huadong Hospital affiliated to Fudan University under the approval number 20,190,030. All patients were informed about the research purposes along with the practical aspects and gave written informed consent prior to inclusion. The trial was registered prior to patient enrollment at Chinese Clinical Trial Registry (http://www.chictr.org.cn; Registration date: 02/03/2019; Identifier: ChiCTR1900021635).

### Study population

Patients were included if they met all the following criteria: (1) scheduled to undergo neurosurgery with an expected duration ≥ 2 h (the reason for choosing neurosurgery is that postoperative chest CT scans could be performed at the same time as routine brain CT scans in patients undergoing neurosurgery); (2) supine position during surgery; (3) age ≥ 18-years-old; (4) American Society of Anesthesiologists (ASA) of I-III; (5) oxygen saturation (SpO_2_) ≥ 94% when breathing room air; (6) body mass index (BMI) < 35 kg/m^2^. The exclusion criteria were as follows: (1) chronic obstructive pulmonary disease; (2) pre-existing atelectasis or pulmonary infection on chest CT scans; (3) obstructive sleep apnea syndrome; (4) heart failure; (5) anticipated difficult intubation; (6) chemotherapy within 3 months; (7) general anesthesia surgery within 1 month. The drop-out criteria included inability to maintain SpO_2_ ≥ 94% during the surgery, operating duration ≤ 2 h, and inability to be extubated. This manuscript adheres to the applicable guidelines of the Consolidated Standards of Reporting Trials (CONSORT) guidelines.

### Randomization and blinding

A stratified block randomization method was conducted, dividing patients into 30% and 60% FiO_2_ groups. As age is an independent risk factor for postoperative atelectasis [19], the trial was stratified by age (< 60 and ≥ 60-years-old). Within each stratum, the participants were randomised at a 1:1 ratio in parallel groups by block randomization with a fixed size of 4. Computer-generated random numbers were implemented by an independent statistician, and allocation with intervention details was sealed in an opaque envelope by an individual not involved in the study.

An anesthesiologist, who was not involved in recruiting patients or collecting outcome data, opened the sealed envelope before the start of anesthesia and provided the designated FiO_2_ setting during mechanical ventilation based on the group assignment. A nurse who was not involved in the study recorded the patient’s vital signs and medication management during the operation. Chest CT was performed by a blinded technician within 30 min after extubation. Postoperative data were collected by a blinded anesthesiologist at 1–3 days after surgery. Throughout the study, the anesthesiologist and nurse in the operating room were aware of group allocation. Patients, clinical researchers, radiologists, technicians, statisticians, and surgical teams were blinded to the allocation information.

### Anesthesia

The participants in the trial followed the standard anesthesia protocol. An arterial catheter was placed into the dorsal artery of the foot under local anesthesia for repeated blood gas sampling and continuous blood pressure monitoring. Propofol, sufentanil, and rocuronium were used for induction of general anesthesia. After tracheal intubation, both groups were ventilated in volume-control mode with a tidal volume of 6–8 ml·kg^− 1^ (predicted body weight) [20], a ventilation rate adjusted to maintain end-tidal CO_2_ between 35 and 45 mmHg, an inspiratory/expiratory ratio of 1:2, and no PEEP or recruitment maneuvers. Anesthesia was maintained with intravenous infusion of propofol and remifentanil.

Neuromuscular blockage was reversed before emergence using neostigmine/anticholinergic agent based on train-of-four ratio stimulation (TOF) monitoring [21]. The patients were extubated after full recovery from the neuromuscular block (TOF ratio ≥ 0.9). After extubation, the patients were transferred to the post-anesthesia care unit (PACU) and supplied with an oxygen face mask with a reservoir.

### FiO_2_ setting

**Fig. 1:**
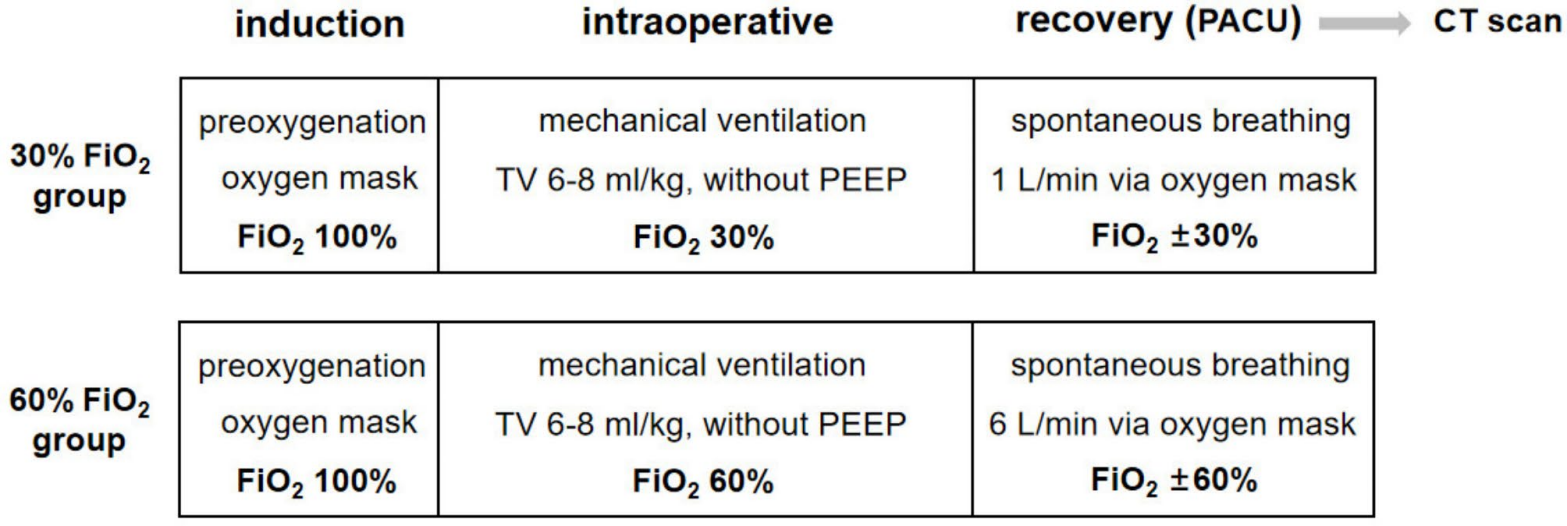
The perioperative management of FiO_2_ in both groups. CT, computed tomography; FiO_2_, inspired oxygen fraction; PACU, post-anesthesia care unit; PEEP, positive end-expiratory pressure; TV, tidal volume

All patients received standard FiO_2_ setting following a detailed protocol. All investigators participating in the study were personally instructed by the principal investigator. During preoxygenation and induction, FiO_2_ was set at 100% in all patients to ensure sufficient oxygen reserves and improve safety when a potentially long period of apnea occurs because of difficulties in airway management. After intubation, the maintenance FiO_2_ was adjusted to 30% or 60% throughout the procedure based on group allocation. After extubation, patients in the 30% or 60% FiO_2_ groups received oxygen at a flow rate of 1 or 6 L/min via an oxygen mask with a reservoir. The FiO_2_ management during the perioperative period is shown in Fig. 1. It should be noted that if the patient’s SpO_2_ < 94% during the operation and in the PACU, the anesthesiologist should increase FiO_2_ or conduct recruitment maneuvers to raise the SpO_2_ to 94–98% [22], proceeding to the withdrawal of the patient.

### Primary outcome

**Fig. 2:**
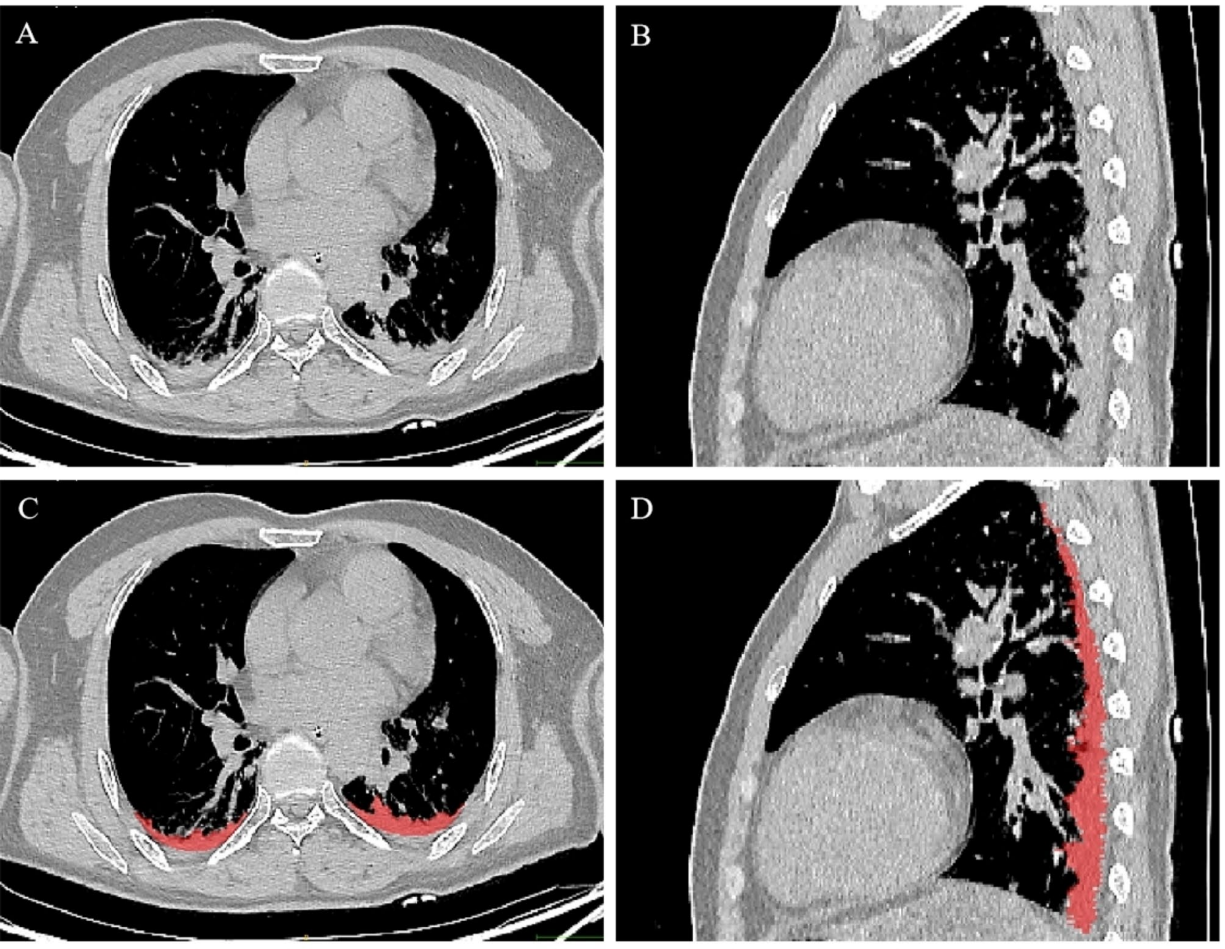
Examples of CT scans. Original chest CT image: (**A**) transaxial and (**B**) sagittal scanning. The red part is the atelectasis area marked by ITK-SNAP software: (**C**) transaxial and (**D**) sagittal scanning. CT, computed tomography

Within 30 min of extubation, the chest CT was performed by a trained and experienced technician who was unaware of the group assignment. All CT images were assessed by an experienced radiologist. The primary outcome was the postoperative atelectasis volume, expressed as a percentage of the total lung volume. The calculation of the percentage of atelectasis volume consisted of three steps. The first step was to measure the total lung area by accurately depicting the contour of the lung image on each CT image with a thickness of 5 mm. The pulmonary hilus vessels were manually excluded from the lung region of interest. The second step was to delineate the volume of the atelectasis region in each CT image. When drawing the atelectasis area, it should be outlined as close to the pleura as possible, and vascular structures with diameters larger than 3 mm should be manually excluded. Lastly, we used the histogram functional view using ITK-SNAP software (version 3.6.0) to identify the atelectasis region (Fig. 2), which was defined as -100 to 100 Hounsfield units [2, 18]. The calculated area was expressed as a percentage of the total lung area in the basal image.

### Secondary outcomes

The percentages of different aeration volumes were considered as secondary outcomes. Areas of different aeration were measured using a workstation software (Sinvo.gia, Siemens Healthcare GmbH) by setting the histogram parameters between − 1,000 and − 901, − 900 and − 501, and − 500 and − 101 Hounsfield Units for over-aeration, normal-aeration, and poor-aeration, respectively [18, 23]. The incidence of clinically important atelectasis, which is defined as a volume of atelectasis of more than 1% lung volume [3], was considered as another secondary outcome. The oxygenation index (PaO_2_/FiO_2_ ratio) before anesthesia and at the end of surgery were considered as secondary outcomes.

### Sample size

Twenty patients were randomly assigned to the 30% or 60% FiO_2_ groups in a pilot study. According to the results of the pilot study, the percentage of postoperative atelectasis volume was 3.56 ± 1.72 in the 30% FiO_2_ group and 4.70 ± 2.44 in the 60% FiO_2_ group. Using the PASS software (version 15.0), setting parameters to α = 0.05 and β = 0.2, the sample size of each group was 55 cases. Further setting the loss to follow-up rate to 10%, the sample size of each group was 60 cases, and the sample size of the two groups combined was 120 cases.

### Statistical analysis

According to the distribution of the data evaluated using the Kolmogorov-Smirnov test, continuous variables were analyzed using the two-sample t-test or Mann-Whitney U test and presented as mean ± standard deviation (SD) or median [interquartile range (IQR)]. Categorical variables were analyzed using the Chi-square test or Fisher’s exact test and reported as numbers and percentages. The primary outcome (the percentage of postoperative atelectasis volume) was normalized using the square root transformation and then analyzed using the two-sample t-test. Moreover, the Mann-Whitney U test was performed to assess the differences in the unnormalized primary outcome data between the two groups. The differences in the oxygenation index at the end of surgery between the two groups were compared, with the oxygenation index before anesthesia as a covariate. A two-sided *P*-value < 0.05 was considered significant for all statistical tests.

We handled missing normalized primary outcome data using multiple imputation by chained equations (MICE), and the iterations were set to 5 [24]. Age, sex, BMI, history of smoking, FiO_2_, and anesthetic duration were used as covariates to impute missing data for multiple imputation. In addition, we performed K-nearest neighbor (KNN) imputation (k = 10, Euclidean distance), regression imputation, and mean imputation as sensitivity analysis to assess the robustness of the primary findings.

## Results

### Subject characteristics

**Fig. 3:**
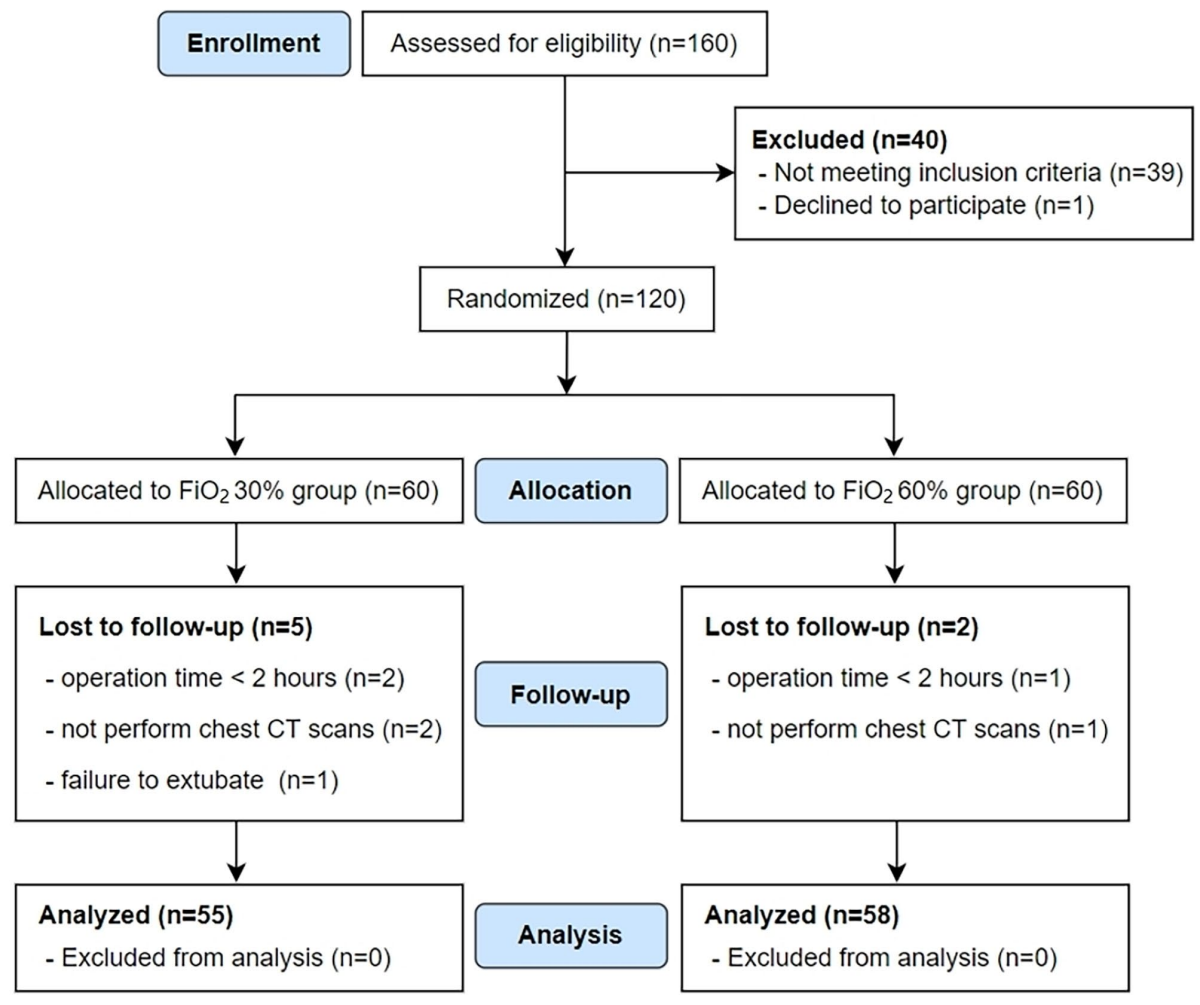
Consolidated Standards of Reporting Trials (CONSORT) diagram. CT, computed tomography; FiO_2_, inspired oxygen fraction

In this study, 120 patients were randomly allocated to either the 30% FiO_2_ group or 60% FiO_2_ groups (Fig. 3). Due to accidental violations of the trial protocol, 7 patients were excluded. Among them, three patients were excluded because of operation time < 2 h (two patients in the 30% FiO_2_ group and one patient in the 60% FiO_2_ group), and three patients were excluded because the did not undergo chest CT scans (two patients in the 30% FiO_2_ group and one patient in the 60% FiO_2_ group). One patient in the 30% FiO_2_ group was also excluded because of failure to be extubated after surgery. No significant differences in patient characteristics and intraoperative data were detected between the two groups (Table 1).

**Table 1:** Patient characteristics and perioperative data

	30% FiO_2_ group (n = 60)	60% FiO_2_ group (n = 60)	*P* value
**Characteristics**			
Age, years	47.9 ± 12.1	48.1 ± 13.0	0.931^a^
Sex, male/female	34/26	36/24	0.711^c^
Education, years	9.0 (6.0, 12.0)	9.0 (9.0, 12.0)	0.525^b^
Height, cm	165.5 ± 7.1	165.2 ± 7.3	0.811^a^
Weight, kg	65.5 (59.3, 72.8)	70.0 (60.0, 74.4)	0.266^b^
BMI, kg/m^− 2^	24.0 ± 2.6	24.9 ± 3.3	0.129^a^
Smoking, n (%)	19 (31.7)	20 (33.3)	0.845^c^
Surgical history, n (%)	24 (40.0)	31 (51.7)	0.200^c^
Hypertension, n (%)	22 (36.7)	23 (38.3)	0.850^c^
Diabetes, n (%)	2 (3.3)	3 (5.0)	1.000^d^
**Intraoperative data**			
Cerebrovascular/Brain tumor	46/14	44/16	0.673^c^
Anesthetic duration, min	211 (169, 274)	202 (152, 293)	0.803^b^
Total liquid intake, mL	1550 (1000, 2238)	1625 (1300, 2000)	0.749^b^
Propofol, mg	1400 (1100, 1800)	1399 (1092, 1784)	0.830^b^
Sufentanil, μg	35 (33, 40)	35 (30, 40)	0.349^b^
Remifentanil, μg	2050 (1673, 2633)	2100 (1587, 2849)	0.910^b^
Time interval between extubation and CT scan, min	35 (35, 45)	37 (29, 44)	1.000^b^

Values are presented as mean ± SD, median (IQR), or n (%). *P* value refers to group comparison of the 30% FiO_2_ group vs. 60% FiO_2_ group by ^a^ two-sample t-test; ^b^ Mann-Whitney U test; ^c^ Chi-square test; ^d^ Fisher’s exact test. BMI, body mass index; FiO_2_, inspired oxygen fraction; IQR, interquartile range; SD, standard deviation

### Primary outcome

There was no significant difference in the percentage of postoperative atelectasis volume between the 30% FiO_2_ group [median (IQR), 3.26 (1.61 to 4.47), n = 55] and the 60% FiO_2_ group [median (IQR), 4.29 (1.83 to 7.27), n = 58, *P* = 0.121] using the Mann-Whitney U test. Moreover, we used square root transformation to normalize the primary outcome, and we also did not find any significant difference in the normalized primary outcome between the 30% FiO_2_ group (mean ± SD, 1.76 ± 0.76, n = 55) and the 60% FiO_2_ group (mean ± SD, 2.02 ± 1.02, n = 58, *P* = 0.124) using a two-sample t-test.

**Fig. 4:**
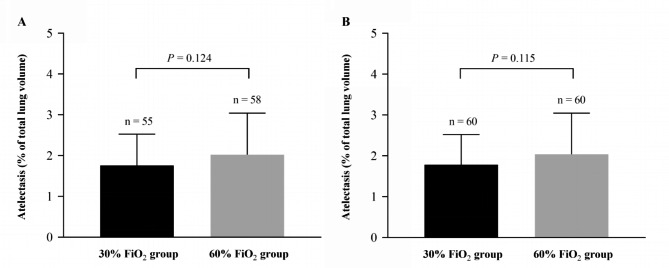
The normalized primary outcome. The percentage of postoperative atelectasis volume is shown by (**A**) original data and (**B**) integrated data of multiple imputation. FiO_2_, inspired oxygen fraction

**Fig. 5:**
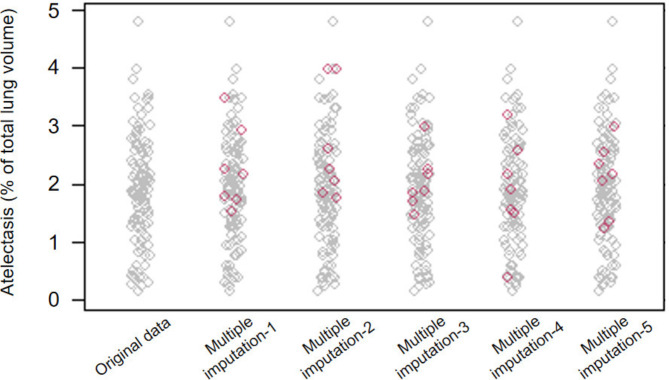
The multiple imputation pattern. Grey square: no missing data; Red square: missing data imputed by multiple imputation

The primary outcome was missing in seven patients among the patients in the randomization, thus we performed multiple imputation to handle missing normalized primary outcome data. Consistent with the results of the original data, none of the five imputations showed significant differences in the percentage of postoperative atelectasis volume between the two groups (Table 2). We further integrated the five imputations datasets and found that there was still no significant difference in the percentage of postoperative atelectasis volume between the two groups (Table 3; Fig. 4). The multiple imputation pattern is shown in Fig. 5.

**Table 2:** The percentage of atelectasis volume handled by multiple imputation (5 imputations datasets)

Multiple imputation	Group	n	mean ± SD	differences (95% CI)	T	*P* value
Imputation − 1	30% FiO_2_	60	1.79 ± 0.75	-0.25 (-0.58, 0.07)	-1.545	0.125
	60% FiO_2_	60	2.04 ± 1.02			
Imputation − 2	30% FiO_2_	60	1.79 ± 0.74	-0.30 (-0.63, 0.03)	-1.786	0.077
	60% FiO_2_	60	2.09 ± 1.06			
Imputation − 3	30% FiO_2_	60	1.79 ± 0.75	-0.23 (-0.56, 0.09)	-1.451	0.150
	60% FiO_2_	60	2.02 ± 1.00			
Imputation − 4	30% FiO_2_	60	1.78 ± 0.78	-0.24 (-0.57, 0.08)	-1.477	0.143
	60% FiO_2_	60	2.02 ± 1.00			
Imputation − 5	30% FiO_2_	60	1.78 ± 0.76	-0.25 (-0.57, 0.07)	-1.543	0.126
	60% FiO_2_	60	2.03 ± 1.00			

CI, confidence interval; FiO_2_, inspired oxygen fraction; SD, standard deviation

**Table 3:** The normalized percentage of atelectasis volume (square root transformation) handled by multiple imputation and sensitivity analysis

Missing value processing method	Group	n	mean ± SD	differences (95% CI)	T	*P* value
Multiple imputation (Integrated)	30% FiO_2_	60	1.79 ± 0.74	-0.26 (-0.57, 0.06)	-1.590	0.115
	60% FiO_2_	60	2.04 ± 1.01			
KNN imputation	30% FiO_2_	60	1.79 ± 0.74	-0.25 (-0.57, 0.07)	-1.557	0.122
	60% FiO_2_	60	2.04 ± 1.00			
Regression imputation	30% FiO_2_	60	1.75 ± 0.74	-0.27 (-0.59, 0.05)	-1.662	0.099
	60% FiO_2_	60	2.02 ± 1.00			
Mean imputation	30% FiO_2_	60	1.76 ± 0.73	-0.26 (-0.58, 0.06)	-1.636	0.105
	60% FiO_2_	60	2.02 ± 1.00			

CI, confidence interval; FiO_2_, inspired oxygen fraction; KNN, K-nearest neighbor; SD, standard deviation

### Sensitivity analysis

KNN imputation, regression imputation, and mean imputation were performed as sensitivity analysis to handle missing normalized primary outcome data (Table 3). No differences in the percentage of postoperative atelectasis volume were observed between the two groups by KNN, regression, or mean imputation, confirming the robustness of our results.

### Secondary outcomes

There were no significant differences in the percentages of over-aeration, normal-aeration, or poor-aeration volumes between the two groups. The overall incidence of clinically significant atelectasis was 83.2%, but again, there was no significant intergroup difference. Additionally, after adjusting for blood gas indicators before anesthesia, the oxygenation index at the end of surgery in the 30% FiO_2_ group did not differ from that in the 60% FiO_2_ group (Table 4).

**Table 4:** Secondary outcomes

Variables	30% FiO_2_ group (n = 55)	60% FiO_2_ group (n = 58)	*P* value
Over-aeration volume, %	9.40 (4.00, 14.30)	9.35 (3.95, 16.80)	0.629^b^
Normal-aeration volume, %	78.00 (72.40, 85.50)	76.30 (69.88, 86.68)	0.389^b^
Poor-aeration volume, %	6.50 (4.70, 9.00)	6.70 (4.55, 9.28)	0.966^b^
Clinically significant atelectasis, n (%)	47 (85.5)	47 (81.0)	0.530^c^
Oxygenation index, mmHg	414.92 ± 81.84	401.50 ± 99.16	0.423^a^

Values are presented as mean ± SD, median (IQR), or n (%). *P* value refers to group comparison of the 30% FiO_2_ group vs. 60% FiO_2_ group by ^a^ two-sample t-test; ^b^ Mann-Whitney U test; ^c^ Chi-square test. FiO_2_, inspired oxygen fraction

## Discussion

In this randomised study, we found no significant differences in the percentage of postoperative atelectasis volume in patients ventilated with 30% FiO_2_ and 60% FiO_2_. In addition, there were also no significant differences in the secondary outcomes, including the different aeration region volumes, incidence of clinically significant atelectasis, and oxygenation index at the end of surgery between the two groups. Taken together, these results suggest that 30% FiO_2_ during mechanical ventilation does not improve postoperative atelectasis compared to 60% FiO_2_.

Recently, a large number of clinical trials have compared the effects of high FiO_2_ (80-100%) versus low FiO_2_ (30-40%) on postoperative atelectasis in patients with mechanical ventilation under general anesthesia, observing a higher incidence of atelectasis in the high FiO2 group [3, 12, 25, 26]. However, it is noteworthy that a moderate FiO_2_ of 50–60% is more conventionally used in clinical practice, while only a few trials have compared moderate FiO_2_ with low FiO_2_. In 2021, Park et al. reported a higher incidence of postoperative atelectasis (39%) in the 60% FiO_2_ group compared with the incidence of atelectasis (20%) in the 35% FiO_2_ group [16]. Whereas in our study, no statistically significant differences in postoperative atelectasis volume were observed between patients applying 30% and 60% FiO_2_. There are several reasons for the contradictory results. First, the FiO_2_ during the postoperative period in the PACU was different between the two studies. Park et al. increased FiO_2_ when patients arrived at the PACU. It has been demonstrated that the use of a high FiO_2_ during the immediate postoperative period in the PACU may carry the potential risk of developing atelectasis [27]. Therefore, we maintained the same FiO_2_ in the PACU as that administered during the intraoperative period. Second, differences in the inclusion population may also have contributed to the differences in the atelectasis between the two groups. The trial by Park et al. included patients undergoing abdominal surgery, most of whom underwent open procedures and were more vulnerable to PPCs. Abdominal surgery may induce elevation of the diaphragm, which may affect pulmonary aeration postoperatively [28–30]. The neurosurgery included in this study could exclude the effect of surgical operation on atelectasis. Third and most importantly, the results may be affected by different detection methods of atelectasis. In the study by Park et al., using lung ultrasound, atelectasis after surgery was detected in 29.7% of the patients. In the present study, we used chest CT to detect atelectasis because it can accurately measure the volume of atelectasis. We found that atelectasis with more than 1% lung volume, which is considered as clinically significant, was detected in 83.2% of patients after surgery. The results were consistent with those of the study by Akca et al. [3] They found that 70% of patients, who received mechanical ventilation during general anesthesia, had postoperative atelectasis with more than 1% lung volume.

In the present study, the areas of different aeration were automatically measured using a workstation software. It was found that there were no significant differences between the two groups regarding the percentages of over-aeration, normal-aeration, and poor-aeration volumes. These results were consistent with the volume of atelectasis. In addition, there was no significant difference in the oxygenation index at the end of surgery between the two groups. The oxygenation index is an index to evaluate the gas exchange in the lung, and it is correlated with atelectasis [31]. Altogether, the results of different aeration volumes and oxygenation index further support the primary findings of the study.

It should be noted that the study is not without limitations. Firstly, the effect of recovery of spontaneous breathing after extubation on atelectasis could not be completely excluded. Secondly, the generalizability of the study is slightly weakened by the mode of mechanical ventilation. In order to explore the effect of intraoperative FiO_2_ on postoperative atelectasis, use of no PEEP/recruitment maneuvers were conducted in the present study, avoiding the interference of other confounding factors during mechanical ventilation. However, the mode of mechanical ventilation (absence of PEEP and recruitment maneuvers) is potentially a promoter of atelectasis [32, 33], which may affect the clinical feasibility of the conclusions of this study. In the future study, we will improve this issue and further explore the comprehensive effects of FiO_2_ and PEEP/recruitment maneuvers on postoperative atelectasis, so as to obatain more meaningful conclusions for clinical practice.

## Conclusions

These results suggest that 30% FiO_2_ does not reduce the volume of postoperative atelectasis compared to 60% FiO_2_ in patients with mechanical ventilation under general anesthesia.

## Data Availability

The datasets used and/or analysed during the current study are available from the corresponding author on reasonable request.

## Abbreviations

ASA: American Society of Anesthesiologists
BMI: Body mass index
CONSORT: Consolidated Standards of Reporting Trials
CT: Computed tomography
FiO_2_: Fraction of inspired oxygen
IQR: Interquartile range
KNN: K-nearest neighbor
MICE: Multiple imputation by chained equations
PACU: Post-anesthesia care unit
PEEP: Positive end-expiratory pressure
PPCs: Postoperative pulmonary complications
SD: Standard deviation
SpO_2_: Oxygen saturation
TOF: Train-of-four stimulation
TV: Tidal volume
WHO: World Health Organization
